# Variability of High-Risk Human Papillomavirus and Associated Factors among Women in Sub-Saharan Africa: A Systematic Review and Meta-Analysis

**DOI:** 10.3390/pathogens12081032

**Published:** 2023-08-11

**Authors:** Michel Carlos Tommo Tchouaket, Aude Christelle Ka’e, Ezechiel Ngoufack Jagni Semengue, Samuel Martin Sosso, Rachel Kamgaing Simo, Bouba Yagai, Alex Durand Nka, Collins Ambe Chenwi, Aissatou Abba, Nadine Fainguem, Carlo-Federico Perno, Vittorio Colizzi, Joseph Fokam

**Affiliations:** 1 Chantal BIYA International Reference Centre for Research on HIV/AIDS Prevention and Management, Yaoundé P.O. Box 3077, Cameroonmartinsosso@yahoo.it (S.M.S.); r.kamgaing@yahoo.it (R.K.S.); romeobouba@yahoo.fr (B.Y.); nkaalexdurand@yahoo.com (A.D.N.); collinschen@yahoo.co.uk (C.A.C.); aichabba@ymail.com (A.A.); fainguem_dine@yahoo.fr (N.F.); 2 School of Health Sciences, Catholic University of Central Africa, Yaoundé P.O. Box 1110, Cameroon; 3 Faculty of Sciences, University of Rome “Tor Vergata”, 00133 Rome, Italy; 4 Faculty of Sciences, Evangelical University of Bandjoun, Bandjoun P.O. Box 127, Cameroon; colizzi@bio.uniroma2.it; 5 Bambino Gesu Pediatric Hospital, 00163 Rome, Italy; cf.perno@uniroma2.it; 6 Faculty of Health Sciences, University of Buea, Buéa P.O. Box 63, Cameroon

**Keywords:** human papillomavirus, human immunodeficiency virus, sub-Saharan Africa, HR-HPV

## Abstract

Background: Sub-Saharan Africa (SSA) carries the highest burden of high-risk human papillomavirus (HR-HPV) in the world, driven by, and together with, HIV infection. This systematic review aimed to identify HR-HPV genotypes and their associated factors among women in SSA. Methods: A systematic review and meta-analysis of studies conducted in SSA on HR-HPV was conducted. Standard electronic databases were searched. R software version 3.6.0 was used for meta-analysis, with *p* < 0.05 considered statistically significant. Results: We included 28 articles with a total of 22,652 participants. The overall pooled prevalence of HR-HPV genotypes was 55.13%, albeit high heterogeneity between studies. The overall pooled prevalence of HR-HPV genotypes in HIV-positive individuals was 75.51%, compared to 52.97% in HIV-negatives (OR = 4.68 (0.71–30.76)). HPV 16 (18%), 35 (10.12%), 52 (9.98%), 18 (9.7%) and 45 (6.82%) genotypes were the most prevalent. Twelve studies identified the most frequently reported risk factors associated with HR-HPV, with HIV infection (66.66%), multiple sexual partners (41.66%) and young age (41.66%) being the most reported risk factors. Conclusions: The combined prevalence of HR-HPV genotypes among women in general and HIV-infected women in particular remains high in SSA. The presence of several genotypes not covered by the vaccine is remarkable and suggests the need for revision of current vaccination policies to prevent HR-HPV infections.

## 1. Background

Human papillomaviruses (HPVs) are viruses responsible for the occurrence of lesions in the anogenital and oropharyngeal regions, which can result in condyloma acuminata, warts that can develop into precancerous lesions and then some years later into cervical cancer [1,2]. Of note, HPV is the most common sexually transmitted infection (STI) in the world, with 660 million people infected according to the World Health Organization [3,4]. Even though the prevalence of cervical cancer varies across geographical settings in the world, in sub-Saharan Africa (SSA) it represents the leading cause of cancer deaths in women [1,5]. Moreover, resource-limited settings (RLS), mainly within SSA, account for more than 80% of cervical cancer deaths, making these settings the most affected areas globally [6,7].

Regarding the pathophysiology, transformation of a cervical lesion into cervical cancer in HIV-negative women takes place over several years, generally induced by a group of so-called high-risk oncogenic viruses (HR-HPV), including the genotypes HPV16, HPV18, HPV31, HPV33, HPV35, HPV39, HPV42, HPV44, HPV45, HPV51, HPV52, HPV53, HPV56, HPV58, HPV59, HPV62, HPV66 and HPV68 [8,9]. These HR-HPVs are very common in young people aged 18 to 30 years, thereby making individuals in this target age range highly vulnerable to acquiring cervical cancers at the population level [10].

In case of co-infection with HIV, the burden of HPV is higher, with more rapid development of cancer from a cervical lesion, in the population of women living with HIV (50.8%) compared to their HIV-uninfected peers (22.6%). This therefore implies a higher vulnerability to HPV and cervical cancer in individuals co-infected with HIV [11,12]. Importantly, several HR-HPV genotypes have been reported among HIV-infected women as compared to their uninfected counterparts (48.4% vs. 17.3% respectively). Taking into consideration the significant burden of HPV in RLS (from 20% to 70%) [13,14] and the prevalence of HIV-infection in these settings (i.e., two-fold higher among women as compared to men), the co-existence of HIV and HPV triggers the pathway toward high-grade cervical squamous intraepithelial lesions (HSIL), which in turn lead to the development of pre-invasive cervical lesions and invasive cervical cancer (ICC) in a substantial population of women living in SSA [15]. This is particularly true as HIV may alter the natural history of HPV infection by decreasing HPV viral clearance, thereby increasing progression to high-grade and invasive lesions, especially in the frame of HR-HPV [16]. HR-HPV prompts integration of viral DNA into the host genome, leading to persistent infection, neoplasia and cervical dysplasia and could ultimately result in cervical cancer [17,18].

Besides HIV infection, several other factors have been reported as predictors of HPV acquisition and vulnerability toward cervical cancer, among which having other STIs (e.g., *Chlamydia*, *Herpes simplex* virus) and multiple sexual partners appear as the major drivers [3,13]. Additionally, factors such as smoking, use of oral or hormonal contraceptives, chronic inflammation and immunosuppressive conditions also favour the acquisition and progression of HPV-induced infection toward cervical oncogenesis. Other factors that may further influence the acquisition/persistence of HPV include dietary habits, socioeconomic status, race/ethnicity and highly burdened geographic location [19,20,21].

According to the WHO report, HPV 16 and 18 genotypes appear to be the most commonly identified in cases of cervical cancer worldwide, alongside other HR-HPV circulating at diverse rates across different geographical settings [22]. However, there is limited evidence from SSA on the genetic diversity of HPV and its pathogenesis, particularly in HIV-infected women [22]. Such studies would enable large-scale mapping of different HPV genotypes in circulation, and the adequacy of existing vaccines on the market, for an optimal evidence-based policy-making for the prevention and management of HPV and cervical cancer in Africa according to specificities of this region. The aim of this study was therefore to conduct a systematic review with meta-analysis of the different HR-HPV genotypes and their association with high-grade cervical dysplasia in SSA.

## 2. Methods

### 2.1. Design

This systematic review with meta-analysis was conducted in accordance with the PRISMA (Preferred Reporting Items for Systematic Review and Meta-Analyses) guidelines [23] and was registered to Prospero under the identification number CRD42021226708.

### 2.2. Data Sources and Search Strategy

**Figure 1:**
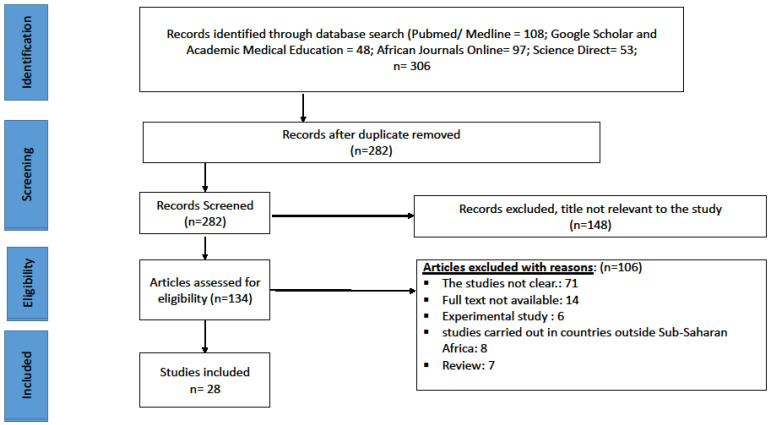
Study flow diagram of studies reviewed, screened and included.

A comprehensive search strategy was applied to PubMed/Medline, Science Direct, African Journals Online, Academic Medical Education and Google Scholar databases to allow for exhaustive identification of relevant studies, using the search terms: “cervical cancer”, “human papillomavirus”, “high-risk human papillomavirus”, “HPV”, “HPV-HR”, “papillomavirus”, “HIV”, “AIDS”, “human immunodeficiency virus”, “acquired immune deficiency syndrome”. In addition, we also included in our search strategy all countries in sub-Saharan Africa, as defined by the United Nations Statistics Division (UNSD) [24]. The search was developed using the Boolean operators “AND” and “OR”. Details of the search are presented in Figure 1. The databases were searched from 1996 to 2021 for studies published in English and French. The reference lists of included articles were also manually searched.

### 2.3. Inclusion and Exclusion Criteria

For this systematic review with meta-analysis, we included: (a) interventional and observational studies published in peer-reviewed journals, including epidemiological surveys, cross-sectional studies, cohorts, case series, case-controls and reports; (b) studies that reported HPV prevalence and/or risk factors; (c) studies with complete HPV genome sequencing results; (d) studies that reported HPV genotyping in HIV-positive/negative women or men and all cases of cervical lesions/cancers; (e) studies that reported the prevalence of HR-HPV or data to calculate this estimate. We defined HR-HPV as the proportion of the number of women with any HPV genotype divided by the total number of women visiting hospitals for lesions of the genital apparatus. For cohort studies, the cumulative incidence was taken as the prevalence, in which the number of new HPV cases was divided by the overall sample size. Case reports, reviews, systematic reviews and meta-analyses, commentaries, studies without full text, sham approach and duplicates were excluded.

### 2.4. Patient and Public Involvement

No patients were involved.

### 2.5. Study Selection and Quality Assessment

An Excel spreadsheet was used to record the studies exported from the online databases. Duplicates identified from the full list of studies were removed. Titles and abstracts of eligible studies were independently reviewed by two authors (M.C.T.T. and A.C.K.) for selection of relevant studies. Differing opinions among investigators regarding study selection were resolved by discussion, consensus and intervention of a third referee if necessary.

The quality of each study was assessed independently by all study authors using a scale dedicated to prevalence studies which is based on 10 components divided into two groups: internal and external validity of the study [25]. Scores of 0 or 1 were assigned to each question in the assessment tool for a total score of 10 per study. Scores of 0–3, 4–6 and 7–10 represented a high, moderate, and low risk of bias, respectively.

### 2.6. Data Extraction

Data from included studies were extracted using a Google form by three authors and verified by M.C.T.T. Data extracted were name of first author, year of publication, study design, inclusion criteria, country, sampling method, study period, age, sex, HIV status, sample size, HPV-HR rate and/or risk factors when identified, and cervical lesion types were extracted. Disagreements observed during data extraction were resolved by discussion and consensus.

### 2.7. Data Analysis

Heterogeneity between studies were estimated by I^2^ and H statistics [25], where I^2^ values indicated the degree of heterogeneity; values of 0%, 18%, 45% and 75% represented none, low, moderate and high heterogeneity, respectively [26]. Lack of evidence on heterogeneity between studies was indicated by obtaining H values close to 1, and these values were inversely correlated with the degree of heterogeneity. Prevalence, 95% confidence intervals (95% CI) and prediction intervals were estimated by random effect models [23,27]. Subgroup analyses according to country and HIV status were employed to adjust variations in the pooled estimate of prevalence. The statistical significance threshold was 0.05. The publication bias was assessed by visual inspection of the asymmetry of the funnel plot [28]. R version 3.6.0 software (packages “meta” and “metafor”) through the RStudio interface was used to perform all meta-analyses [29,30].

## 3. Results

### 3.1. Study Characteristics

**Figure 2:**
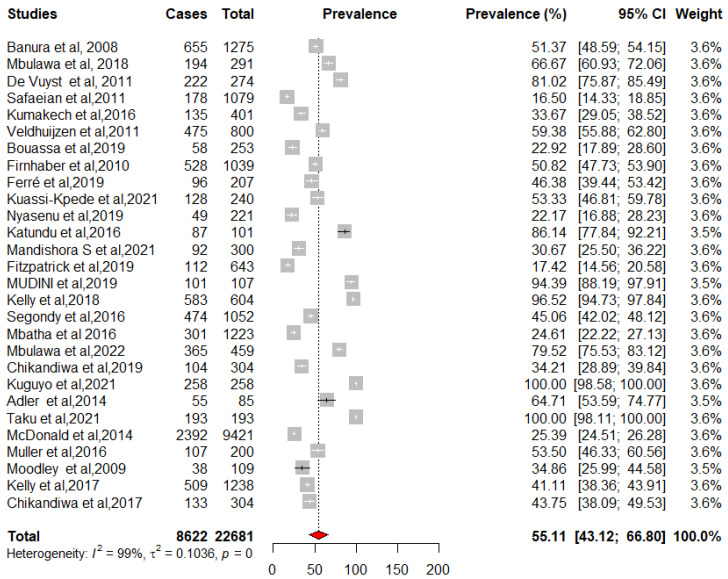
Forest plot to estimate the combined prevalence of human papillomavirus infection in women with 95% CI (weighted estimate based on the random effects model): ES—prevalence equivalent effect size, CI—confidence interval. In the graph, the diamond indicates the pooled result and the boxes indicate the effect estimates of the individual studies. The dotted purple vertical line indicates the pooled estimate. The solid purple vertical line indicates the baseline at zero indicating no effect. The horizontal line through the boxes shows the length of the confidence interval and the boxes show the effect estimates from the individual studies [3,27,29,31,32,33,34,35,36,37,38,39,40,41,42,43,44,45,46,47,48,49,50,51,52,53,54,55].

We selected 28 studies for this systematic review (Figure 1 and Figure 2). One study was conducted in Kenya [31], three in Uganda [3,32,33], one in Rwanda [34], one in Chad [35], three in Togo [36,37,38] (we included one study conducted in men as it met our eligibility criteria), one in Zambia [39], four in Zimbabwe [40,41,42,43], two in Burkina Faso [44,45] and finally 14 in South Africa [44,46,47,48,49,50,51,52,53]. It should be noted that two studies were conducted simultaneously in two countries, namely Burkina Faso and South Africa in one study and Kenya and South Africa in the other. Participants in these studies were recruited from urban and rural areas, and samples were collected and analysed in health facilities (hospital, clinic, research laboratory).

According to the type of study found, we were able to retain 28 studies for our review, distributed as follows: 18 cross-sectional studies; 9 cohort studies and 1 case-control study. The articles included in this systematic review with meta-analysis were published between 2008 and 2022. Based on the sample size of the included studies, the minimum sample size was 85 participants and the maximum sample size was 8622 participants. The age of the participants in the different studies ranged from 15 to 90 years. Thirteen studies did not indicate the age range of the participants (Table 1).

### 3.2. Pooled Prevalence of HR-HPV

**Figure 3:**
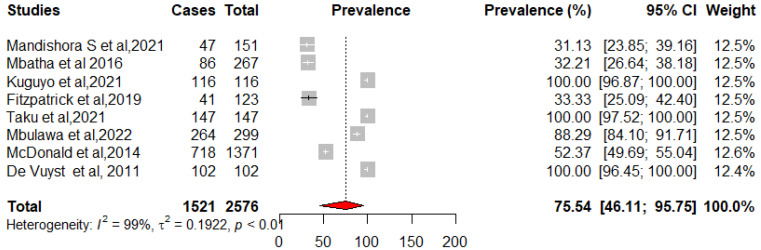
Forest plot for estimating the combined prevalence of high-risk human papillomavirus infection among HIV-infected participants (weighted estimate based on the random effects model): ES—prevalence equivalent error size, CI—confidence interval. In the graph, the diamond indicates the pooled result and the boxes indicate the effect estimates of the individual studies. The dotted purple vertical line indicates the pooled estimate. The solid purple vertical line indicates the baseline at zero indicating no effect. The horizontal line through the boxes shows the length of the confidence interval and the boxes show the effect estimates from the individual studies [31,40,41,42,46,47,50,51].

**Figure 4:**
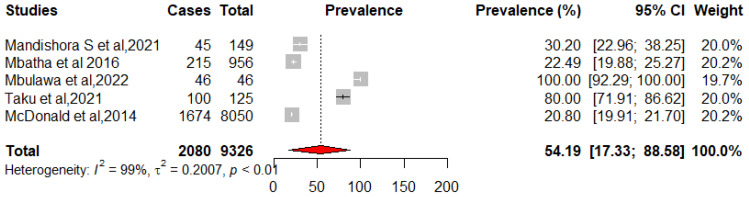
Forest plot for estimating the combined prevalence of high-risk human papillomavirus infection in HIV-positive women (weighted estimate based on the random effects model): ES—prevalence equivalent error size, CI—confidence interval. In the graph, the diamond indicates the pooled result and the boxes indicate the effect estimates of the individual studies. The dotted purple vertical line indicates the pooled estimate. The solid purple vertical line indicates the baseline at zero indicating no effect. The horizontal line through the boxes shows the length of the confidence interval and the boxes show the effect estimates from the individual studies [40,46,47,50,51].

**Figure 5:**
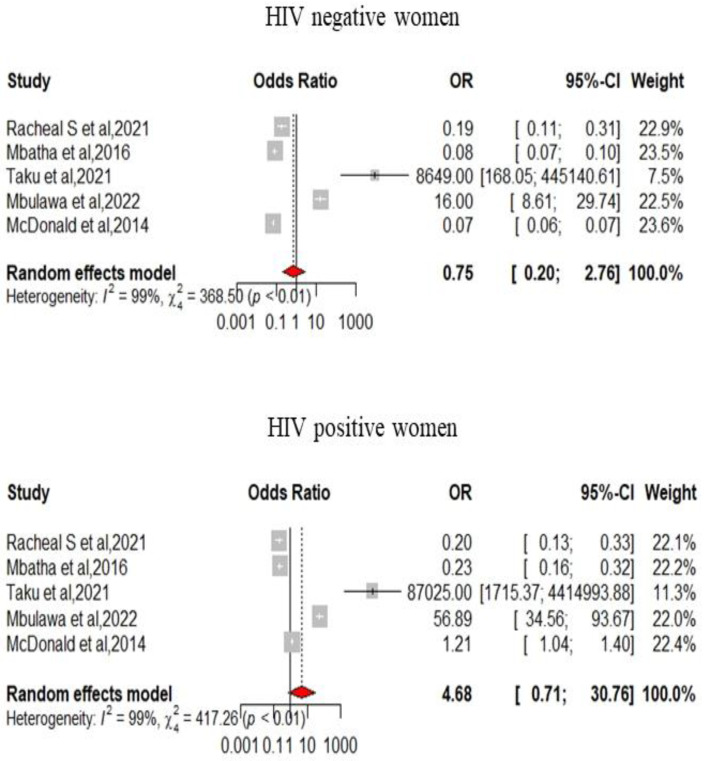
Forest plot for estimating the combined prevalence of high-risk human papillomavirus infection among HIV+/HIV− infected women (weighted estimate based on the random effects model): ES—prevalence equivalent error size, CI—confidence interval. In the graph, the diamond indicates the pooled result and the boxes indicate the effect estimates of the individual studies. The dotted purple vertical line indicates the pooled estimate. The solid purple vertical line indicates the baseline at zero indicating no effect. The horizontal line through the boxes shows the length of the confidence interval and the boxes show the effect estimates from the individual studies [40,46,47,50,51].

We pooled data from 22,652 HIV-infected women to estimate the pooled prevalence of HPV infection using a meta-analysis. The overall pooled prevalence of all HR-HPV genotypes was 55.11% (95%CI: 43.12–66.80) (Figure 2) with high heterogeneity between studies [x^2^ = 0.0719 (NS = 28); *p*-value = 0 and I^2^ = 99%]. The overall pooled prevalence of HR-HPV genotypes in HIV+ individuals was 75.51% [95% CI: 51.35–93.30] (Figure 3) with high heterogeneity between studies [x^2^ = 0.1313 (NS = 8); *p*-value < 0.01 and I^2^ = 99%] and a significant difference in prevalence reported across included studies. The overall pooled prevalence of HR-HPV genotypes in HIV-negative individuals was 54.19% [95% CI: 17.33–88.58] (Figure 4) with high heterogeneity between studies [x^2^ = 81.52 (NS = 6); *p* value < 0.01 and I^2^ = 99%], with a significant difference in prevalence reported across included studies (Figure 5).

The pooled prevalence of HR-HPV genotypes was also estimated in the studies (i.e., genotypes 16, 18, 31, 33, 35, 39, 45, 51, 52, 56, 58, 59, 61, 62, 66 and 68). The observed genotypes are classified as follows in order of prevalence: HPV16 (18%), HPV35 (10.12%), HPV52 (9.98%), HPV18 (9.7%), HPV45 (6.82%), HPV51 (6.60%), HPV58 (6.40%), HPV56 (6.20%), HPV33 (6.10%), HPV31 (5.90%), HPV39 (4.20%), HPV68 (3.96%), HPV59 (3.41%), HPV66 (2.20%), HPV62 (0.30%) and HPV61 (0.10%). Remarkably, most of these identified genotypes are not yet included in vaccines, particularly in those available in African countries. Most countries in sub-Saharan Africa have easy access to the bivalent and quadrivalent vaccines due to limited resources or lack of evidence for the 9-valent vaccines regularly used in developed countries (Table 2). 

### 3.3. Subgroup Analysis

**Figure 6:**
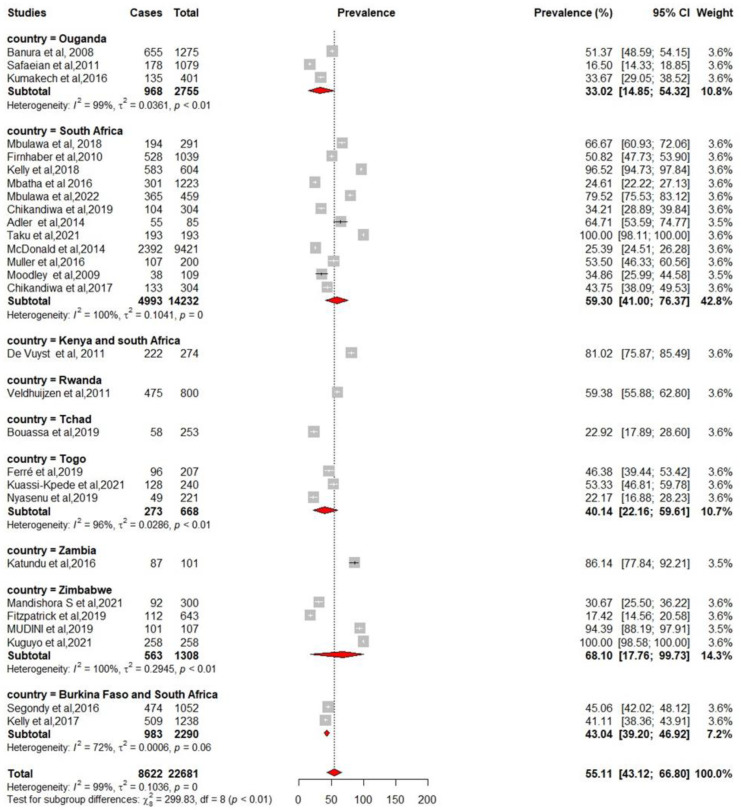
Forest plot for estimating the combined prevalence of high-risk human papillomavirus infection among women by country in sub-Saharan Africa (weighted estimate based on the random effects model): ES—prevalence equivalent error size, CI—confidence interval. In the graph, the diamond indicates the pooled result and the boxes indicate the effect estimates of the individual studies. The dotted purple vertical line indicates the pooled estimate. The solid purple vertical line indicates the baseline at zero indicating no effect. The horizontal line through the boxes shows the length of the confidence interval and the boxes show the effect estimates from the individual studies [3,29,31,33,34,35,37,38,39,40,41,42,43,44,46,47,48,49,50,51,54,55].

The result of the subgroup analysis of sub-Saharan Africa from which the studies were drawn shows significant heterogeneity between and within some countries. For example, the pooled prevalence of HR-HPV in Zimbabwe was 68.10% [95% CI: 16.65–99.89] with a heterogeneity of (x^2^ = 0.3109 (NS = 04); *p*-value < 0.01 and I^2^ = 100%). The pooled prevalence of HR-HPV in South Africa was 59.42% [95% CI: 41.26–76.36] with a heterogeneity of (x^2^ = 0.1025 (NS = 12); *p*-value = 0 and I^2^ = 100%). In Uganda, the pooled prevalence of HR-HPV was 33.02% [95% CI: 12.27–58.05] with a heterogeneity of (x^2^ = 0.0499 (NS = 03); *p*-value < 0.01 and I^2^ = 99%). In Togo, the pooled prevalence of HR-HPV was 40.14% [95% CI: 22.05–59.74] with a heterogeneity of (x^2^ = 0.0290 (NS = 03); *p*-value < 0.01 and I^2^ = 96%). Data from Burkina Faso and South Africa revealed a pooled prevalence of 43.04% [95% CI: 39.20–46.92] with a heterogeneity of (x^2^ = 0.0006 (NS = 02); *p*-value = 0.06 and I^2^ = 72%); while four countries had no applicable heterogeneity (Figure 6).

### 3.4. Laboratory Methods to Detect HR-HPV Infection from the Included Studies

The molecular genotyping and HR-HPV detection techniques applied in the selected studies were as follows: next generation sequencing (95% sensitivity and 99.99% specificity; storage temperature: −20 °C to 25 °C; manual interpretation of results, two days for analysis delay), Linear Array HPV Genotyping Test (97% sensitivity and 90.5% specificity; storage temperature: −20 °C to 25 °C; automatic interpretation of results, 8 h for analysis delay), HPV care, genotyping by PCR restriction fragment length polymorphism analysis (with 70% sensitivity and 90% specificity; storage temperature: −20 °C to 25 °C; manual interpretation of results, 12 h for analysis delay), slide-based reverse hybridisation, multiplex RT-PCR, Sacace Biotechnologies, Anyplex II HPV28 real-time PCR (70% sensitivity and 90% specificity; storage temperature: −20 °C to 25 °C; automatic interpretation of results, 12 h for analysis delay), The Hybrid Capture DNA 2 (HC2) test (97% sensitivity and 97% specificity), INNO-LiPA HPV genotyping Extra^®^ test (97% sensitivity and 90.5% specificity; storage temperature: −20 °C to 25 °C; automatic interpretation of results, 8 h for analysis delay) (Table 1). The varying performances of these HPV diagnostic tools suggest some inter-assay disparities and the need to develop adapted algorithms according to available tools and circulating HPV-genotypes within the setting of interest. Furthermore, these tests need a certain number of devices to be installed, they use expensive kits that cannot be stored at room temperature, interpretation of results is automatic for some platforms and manual for others, and use may require qualified personnel [55].

### 3.5. Factors Associated with HR-HPV Infection from the Included Studies

HIV infection, a history of having sex with three or more sexual partners, young age, condom use, sexually transmitted infection, single marital status, precarious employment, use of hormonal contraceptives, alcohol consumption and tobacco use were the most frequently reported risk factors associated with HR-HPV infection (Table 1).

### 3.6. Prevalence of HPV Infection in HIV+ Women When Compared to HIV− Women

Five studies reported the rate of HPV positivity in HIV-positive and HIV-negative women respectively. In HIV-negative women, the rate of HPV positivity was 29.97% (3295/10,991), while the rate of HR-HPV positivity was 61.72% (1751/2837), with HIV-infection increasing by more than four times the risk of being infected by HR-HPV (OR = 4.68 [0.71–30.76]) (Figure 5).

### 3.7. Cervical Lesions and HR-HPV Infection in the Included Studies

Nine studies reported data on the cervical lesion status of participants in their studies; in total, 3814 tests were performed, and 459 participants had low grade squamous intraepithelial lesion (LSIL) compared to 534 who had high grade squamous intraepithelial lesion (HSIL). Two studies reported that the HR-HPV genotypes most commonly found in cervical lesions in the selected studies were 16 and 35 (Table 3).

## 4. Discussion

The objective of this study was to conduct a systematic review with meta-analysis to identify the different HR-HPV genotypes and their relationship with HIV, to verify their association with high-grade cervical dysplasia in sub-Saharan Africa, and to verify their adequacy with available vaccines. Thus, the synthesis of studies with detailed data on HIV-infected women showed that the prevalence of HR-HPV is high but similar to the prevalence of HR-HPV in HIV-uninfected women; this observed difference seems surprising. However, this result may also reflect the success of antiretroviral treatment (ART) among people living with HIV(PLHIV) due to scale-up of ART, which in turn ensures immune reconstitution to normalcy at level similar to that of HIV-negative individuals [56].

We decided to include studies that used a complete diagnostic kit capable of demonstrating at least eight HPV-HR genotypes. Although these kits are very costly for resource-limited countries and require state-of-the-art technical facilities with qualified technicians, they have the advantage of demonstrating the HPV-HR genotypes that are prevalent in a region, and this information could have an impact on the appropriate vaccine for sub-Saharan Africa to eradicate this virus responsible for several public health problems [54].

HIV infection, a history of sex with three or more partners, young age of the woman, smoking, use of hormonal contraceptives were found to be the most frequently associated risk factors in this study. Smoking is known to act as an immunosuppressant, which decreases the host immune response to invasion of an HR-HPV genotype. With regard to sexuality, early sexual debut is an important risk factor, partly because of the immaturity of the cervix during adolescence, which makes it more susceptible to infection and damage by HR-HPV, and several authors have already shown that having sex with multiple partners significantly increases the possibility of exposure to HR-HPV [7,27].

With a positivity rate of 55.13% across all included studies, several high-risk oncogenic genotypes were revealed (genotypes HPV16, 18, 31, 33, 35, 39, 45, 51, 52, 56, 58, 59, 61, 62, 66 and 68), with the most prevalent being HPV16 (the highest, 18.00%), followed by HPV35 (10.12%), HPV52 (9.98%) and HPV18 (9.87%). With the variability of genotypes identified from country to country, according to data generated by researchers in sub-Saharan Africa, it is essential to have data from each region in order to develop a good policy for the prevention of genital HPV infections which are significantly prevalent in the general population and in PLHIV in particular, who remain a vulnerable population when not put on ART [56]. Similar results were reported in a meta-analysis conducted in Ethiopian women, where genotypes HPV16, 52, 35, 18, 58, 51, 45, 31, 53 and 56 (in decreasing order of prevalence) were the ten most common genotypes in this sub-Saharan African country. This dense genetic diversity in a context of non-adherence of the population to vaccination could be one of the reasons for the increase in the positivity rate of circulating HR-HPV in resource-limited countries [57]. The persistently high rate of HR-HPV positivity means that transmission of the infection continues to increase, highlighting the unsuspected high burden of cervical HR-HPV infection among women in sub-Saharan Africa, and underscoring the importance of implementing preventive measures such as vaccination to prepare the immune systems of these women to fight these infections [58]. Based on the information collected, our observations suggest that a large majority of these HR-HPV infections could a priori have been prevented by the use of Gardasil-9 vaccine (covering nine different genotypes, namely HPV6, 11, 16, 18, 31, 33, 45, 52, 58), which could provide much better vaccine coverage in sub-Saharan Africa. However, for a variety of reasons (which could also be budgetary or scarcity of evidence-based findings), the current vaccination strategy available in most sub-Saharan countries that have adopted the vaccine is based on Gardasil 4 (targeting genotypes 6, 11, 16 and 18) or Cervarix (targeting genotypes 16 and 18). Hence, our current finings strongly support the introduction of Gardasil-9 vaccine into the national immunization program of sub-Saharan African countries for an optimal control of the increasing prevalence of HR-HPV genotypes in Africa. At the same time, and despite this great implementation of coverage by Gardasil-9, our study shows that nine other high-risk genotypes are not covered by Gardasil-9 vaccine, with genotypes such as HPV-35, HPV-51 and HPV-56 sometimes having a higher prevalence than the vaccine genotypes, as reported in Chad [35,59].

The association between HIV and HPV is well documented, with HIV increasing the risk of HPV infection and persistence [34]. Furthermore, summary estimates generated within this work revealed that HIV-infected women are more than four times at risk to be infected by HR-HPV in sub-Saharan Africa than their HIV-negative peers, in line with previous findings in this sub-region and worldwide [60,61]. Although adolescents have a low risk of cervical cancer, HIV-positive adolescents have a high risk of abnormal cervical cytology and are more likely to have persistent HPV infections [39]. In addition, the immune response induced by the quadrivalent HPV vaccine also appears to be robust in HIV-positive women, even as vaccine recommendations are identical in both groups. Modelling studies estimate that with adequate coverage, vaccination with the HPV16/18 vaccine could prevent 36–45% of invasive cervical cancers in countries such as Zambia [36].

All of the genotypes identified, especially those not yet included in the literature review, were made possible by the use of comprehensive molecular diagnostic techniques, which are increasingly available to laboratories in sub-Saharan Africa, thanks to collaboration between researchers, institutions and organisations in the North and South [22]. Authors have joined forces to promote science, which has led to the transfer of knowledge and skills, the reduction of boundaries, the mitigation of costs, the enhancement of the benefits of research results and the measurement of attributes. This justifies the interest of researchers to do more work on this topic of great importance to public health in sub-Saharan Africa [62].

Very few studies have reported data on the status of cervical lesions during infection and other coinfections. The available data nonetheless confirm that HIV is a major risk factor for the development of LSILs and HSILs, although the relative impact on HSILs is smaller [63]. This is consistent with cohort studies which show that, although HIV-positive women have a high number of cytological abnormalities, the vast majority of lesions are low grade, with only a slight increase in the prevalence of HSIL [64]. Therefore, HIV-positive women may be overtreated in screening programs due to the high number of low-grade lesions that may progress to HSIL [65].

In brief, it appears from our findings that accessibility to HPV screening tools needs to be expanded both within health facilities and at the community level. An optimal diagnostic algorithm, adapted to the context also needs to be developed and validated in low-income settings like sub-Saharan Africa, accompanied with training of health professionals, timely diagnosis and appropriate monitoring of women living with HIV/HPV co-infection as a vulnerable population. We must also enhance the sensitisation of stakeholders and community engagement for adherence to HPV screening and prevention strategies.

## 5. Conclusions

This systematic review indicates that the rate of HR-HPV genotype positivity among women in general and in HIV-infected women in particular remains high in sub-Saharan Africa, with no significant disparity compared to their HIV-negative peers, probably due to widespread access to antiretroviral treatment (“test and treat” strategy) aimed at eradicating this pandemic. The study also showed that an alarming number of HR-HPV genotypes are circulating in sub-Saharan Africa, with a considerable presence of genotypes not covered by the vaccination policy implemented by the health systems of certain countries, calling into question existing public health policies against cervical cancer. Cervical cancer diagnosis and treatment programs therefore need to be strengthened, while vaccination policies against new emerging HR-HPV genotypes need to be implemented in these contexts.

## Figures and Tables

**Table 1:** Molecular genotyping techniques and associated factors for HPV infection.

	Author, Year	Molecular Genotyping Technique/(Targeted Genotypes)	Sample Size	Number with HR-HPV+	Risk Factors Associated with HR-HPV Positivity
1	Banura et al., 2008 [3]	Test HPV Linear Array de Roche/(17-HPV)	1275	762	HIV infection, employment in the service sector, multiple sexual partners
2	Mbulawa et al., 2018 [27]	Genotyping Roche Linear Array HPV/(37-HPV)	291	194	STI; multiple sexual partners
3	De Vuyst et al., 2011 [31]	PCR-HPV by in-line reverse hybridization (14-HPV)	274	235	Multiple sexual partners and age
4	Safaeian et al., 2008 [32]	Test Roche-PCR line blot/(13-HPV)	1079	264	Age; HIV infection; single women, two or more partners in the past year.
5	Kumakech et al., 2016 [33]	Genotyping test HPV, CLART^®^ HPV2/(35-HPV)	401	70	-
6	Veldhuijzen et al., 2011 [34]	Genotyping test HPV Linear Array/(37-HPV)	800	487	Hormonal contraceptives and age
7	Bouassa et al., 2019 [35]	Detection kit Anyplex II HPV28 (28-HPV)	253	56	Multiple sexual partners
8	Firnhaber et al., 2010 [36]	Roche Linear Array HPV/(37-HPV)	1010	528	-
9	Ferré et al., 2019 [37]	Detection kit Anyplex II HPV-28/(28-HPV)	207	107	-
10	Kuassi-Kpede et al., 2021 [38]	RT-PCR multiplex, Sacace Biotechnologies (14-HPV)	240	207	-
11	Nyasenu et al., 2019 [39]	Kit PCR Mix and Phire Hot Start II/(36-HPV)	221	52	HIV infection, age
12	Katundu et al., 2016 [40]	Linear Array genotyping assay (Roche)/(37-HPV)	101	89	-
13	Mandishora et al., 2021 [41]	Next generation sequencing	300	97	HIV infection
14	Fitzpatrick et al., 2019 [42]	Detection kit Anyplex II HPV HR/(14-HPV)	643	111	-
15	Mudini et al., 2019 [43]	MY09/MY11 PCR and hybridisation typing/(37-HPV)	107	104	-
16	Kelly et al., 2018 [44]	Genotyping INNO-LiPA HPV Extra/(37-HPV)	604	809	-
17	Segondy et al., 2016 [45]	Genotyping test INNO-LiPA HPV Extra (28-HPV)	1052	422	HIV infection, marriage, alcohol, age, tobacco
18	Mbatha et al., 2016 [46]	Roche Linear Array HPV genotyping test/(18-HPV)	1223	482	-
19	Mbulawa et al., 2018 [47]	Genotyping test Roche Linear Array/(37-HPV)	459	371	-
20	Chikandiwa et al., 2019 [29]	Roche Linear Array (RLA, Roche Diagnostics)/(37-HPV)	304	104	-
21	Kuguyo et al., 2021 [41]	Detection kit Anyplex ™ II HPV HR/(14-HPV)	258	382	-
22	Adler et al., 2014 [49]	Test HPV Linear Array de Roche/(13-HPV)	85	64	-
23	Taku et al., 2021 [50]	Direct flow HPV kit/(36-HPV)	193	300	HIV infection
24	McDonald et al., 2014 [51]	Hybrid capture DNA test 2 (HC2)/(13-HPV)	9421	1994	-
25	Muller et al., 2016 [52]	Roche HPV Linear Array (37-HPV)	200	107	-
26	Moodley et al., 2006 [53]	Test Digene Hybrid Capture 2 (HC2) (13-HPV)	109	38	-
27	Kelly et al., 2017 [54]	Test INNO-LiPA HPV/(37-HPV)	1238	467	HIV infection
28	Chikandiwa et al., 2017 [55]	PCR system GeneAmp 9700 de Roche/(37-HPV)	304	133	HIV infection, multiple sexual partners, condoms
	Total		22,652	9036	

**Table 2:** Prevalence of different HPV genotypes and country included in the meta-analysis of women.

	Author and Year	Country	HPV 16	HPV 18	HPV 31	HPV 33	HPV 35	HPV 39	HPV 45	HPV 51	HPV 52	HPV 56	HPV 58	HPV 59	**HPV** **61**	**HPV** **62**	**HPV** **66**	**HPV** **68**	**Total**
1	Banura et al., 2008 [3]	Ouganda	93	105	54	83	42	34	21	92	105	65	20	19	-	-	-	29	762
2	Mbulawa et al., 2022 [27]	South Africa	43	27	25	-	-	-	-	31	-	-	39	-	-	-	29	-	194
3	De Vuyst et al., 2011 [31]	Kenya and South Africa	118	37	3	6	17	2	33	1	-	5	7	1	-	-	-	5	235
4	Safaeian et al., 2011 [32]	Ouganda	32	18	10	17	13	18	23	37	22	20	14	22	-	-	-	18	264
5	Kumakech et al., 2016 [33]	Ouganda	0	4	7	1	5	5	2	13	19	-	-	-	-	-	14	-	70
6	Veldhuijzen et al., 2011 [34]	Rwanda	29	20	39	34	32	40	37	48	50	34	60	28	-	-	-	36	487
7	Bouassa et al., 2019 [35]	Tchad	5	4	6	1	6	3	5	-	5	6	7	-	-	4	4	-	56
8	Ferré et al., 2019 [36]	Togo	27	-	26	-	31	-	-	-	-	-	-	23	-	-	-	-	107
9	Kuassi-Kpede et al., 2021 [37]	Togo	3	18	24	3	22	7	10	25	24	29	12	7	-	-	12	11	207
10	Nyasenu et al., 2019 [38]	Togo	3	20	-	-	-	-	-	-	-	-	-	-	-	11	-	18	52
11	Katundu et al., 2016 [39]	Zambia	24	24	-	-	-	-	3	18	-	-	20	-	-	-	-	-	89
12	Mandishora et al., 2021 [40]	Zimbabwe	20	7	-	-	-	7	10	-	18	-	14	-	-	9	5	7	97
13	Kuguyo et al., 2021 [41]	Zimbabwe	123	65	17	25	68	10	14	11	9	6	23	3	-	-	1	7	382
14	Fitzpatrick et al., 2019 [42]	Zimbabwe	12	12	5	6	12	2	10	6	12	6	10	4	-	-	3	11	111
15	Mudini et al., 2019 [43]	Zimbabwe	59	20	-	7	-	-	4	-	-	9	5	-	-	-	-	-	104
16	Kelly et al., 2018 [44]	South Africa	115	89	61	48	99	48	47		146	58	54	11	-	-	-	33	809
17	Segondy et al., 2016 [45]	Burkina Faso and South Africa	41	33	49	16	36	21	20	23	83	25	22	3	-	-	27	23	422
18	Mbatha et al., 2016 [46]	South Africa	99	56	-	47	50	15	34	58	32	42	15	31	-	-	-	3	482
19	Mbulawa et al., 2018 [47]	South Africa	133	44	-	-	67	-	44	-	37	-	46	-	-	-	-	-	371
20	Chikandiwa et al., 2019 [29]	South Africa	18	9	4	4	4	2	15	8	5	5	10	12	-	-	-	8	104
21	Firnhaber et al., 2010 [48]	South Africa	208	-	-	71	-	-	-	-	-	112	-	56	-	-	81	-	528
22	Adler et al., 2014 [49]	South Africa	10	7	1	1	6	2	9	6	6	2	2	2	-	-	-	10	64
23	Taku et al., 2021 [50]	South Africa	40	17	22	25	44	12	29	13	26	16	32	-	-	-	16	8	300
24	McDonald et al., 2014 [51]	South Africa	247	179	129	132	279	100	194	157	174	112	126	68	-	-	-	97	1994
25	Muller et al., 2016 [52]	South Africa	48	7	5	-	3	-	10	34	-	-	-	-	-	-	-	-	107
26	Moodley et al., 2009 [53]	South Africa	6	5	-	-	2	3	5	-	-	-	2	-	8	-	7	-	38
27	Kelly et al., 2017 [54]	Burkina Faso and South Africa	51	42	47	19	62	43	26	-	121	-	27	6	-	-	-	23	467
28	Chikandiwa et al., 2017 [55]	South Africa	19	10	3	6	15	6	12	13	8	7	10	13	-	-	-	11	133
Total *n* (%)			1626 18%	879 9.7%	537 5.9%	552 6.1%	915 10.1%	380 4.2%	617 6.8%	594 6.6%	902 9.9%	559 6.2%	577 6.4%	309 3.4%	8 0.1%	24 0.3%	199 2.2%	358 3.9%	9036 100%

**Table 3:** Cervical vaginal smear and HR-HPV infection.

	Author Year	Sample Size	Sample Size of Cervical Vaginal Smear	Number with HR-HPV+	Cervical Cancer
1	Banura et al., 2008 [3]	1275	-	-	-
2	Mbulawa et al., 2018 [27]	291	-	-	-
3	De Vuyst et al., 2011 [31]	274	-	-	-
4	Safaeian et al., 2011 [32]	1079	-	-	-
5	Kumakech et al., 2016 [33]	401	-	-	-
6	Veldhuijzen et al., 2011 [34]	800	-	-	-
7	Bouassa et al., 2019 [35]	253	-	-	-
8	Ferré et al., 2019 [36]	207	-	-	-
9	Kuassi-Kpede et al., 2021 [37]	240	-	-	-
10	Nyasenu et al., 2019 [38]	221		-	-
11	Katundu et al., 2016 [39]	101	-	-	-
12	Mandishora et al., 2021 [40]	300	-	-	-
13	Kuguyo et al., 2021 [41]	258	-	-	-
14	Fitzpatrick et al., 2019 [42]	643	76	112	Normal = 44 LSIL = 16 HSIL = 16
15	Muduni et al., 2019 [43]	107	-	-	-
16	Kelly et al., 2018 [44]	604	-	-	-
17	Segondy et al., 2016 [45]	1052	426	422	Normal = 368 LSIL = 33 HSIL = 25
18	Mbatha et al., 2016 [46]	1223	-	-	-
19	Mbulawa et al., 2018 [47]	459	272 *	371 *	16 and 35 were the most dominant genotypes in HSIL *
20	Chikandiwa et al., 2019 [29]	304	-	-	-
21	Firnhaber et al., 2010 [48]	1010	926	528	Normal = 507 LSIL = 237 HSIL = 182
22	Adler et al., 2014 [49]	85	85	64	Normal 69; LSIL 8; HSIL = 8
23	Taku et al., 2021 [50]	193	193	300	Normal = 0 LSIL = 43 HSIL = 147
24	McDonald et al., 2014 [51]	9421	1848 *	1994 *	16 and 35 were the most dominant genotypes in HSIL *
25	Muller et al., 2016 [52]	200	-	-	-
26	Moodley et al., 2009 [53]	109	109	38	Normal = 20 LSIL = 40 HSIL = 49
27	Kelly et al., 2017 [54]	1238	-	-	-
28	Chikandiwa et al., 2017 [55]	304	298	133	Normal = 0 LSIL = 137 (46) HSIL = 161 (87)
	Total	22,652	2113	1597	

* Studies with precise estimates on genotype distribution.

## Data Availability

The dataset is available from the corresponding author.

## Abbreviations

AIDS	Acquired immunodeficiency syndrome
CC	Cervical Cancer
CIRCB	“Chantal BIYA” International Reference Centre for research on HIV/AIDS prevention and management
HIV	Human Immunodeficiency Virus
HPV	human papilloma virus
HR-HPV	high risk oncogenic Human Immunodeficiency Virus
HSIL	high-grade intraepithelial lesion
ICC	Invasive Cervical Cancer
LR-HPV	Low Risk non-Human Papilloma Virus
LSIL	low-grade intraepithelial lesion
PLHIV	People Living with HIV
RT-PCR	Real-Time Polymerase Chain Reaction
UNSD	United Nations Statistic Division
